# Soft-tissue thickness radiographic measurement: a marker to evaluate acute periprosthetic joint infection risk in total hip replacement

**DOI:** 10.5194/jbji-6-211-2021

**Published:** 2021-06-04

**Authors:** Laura Rey Fernández, Francesc Angles Crespo, Silvia María Miguela Álvarez, Martí Carles Bernaus-Johnson, Agustí Bartra Ylla, Lluís Font-Vizcarra

**Affiliations:** 1 Department of Traumatology and Orthopaedic Surgery, Hospital Universitari Mútua Terrassa, Barcelona, Spain; 2 Department of Surgery, Universitat de Barcelona, Barcelona, Spain

## Abstract

The objective of our study was to evaluate the association between acute periprosthetic joint infection (APJI) and radiographic measurement of soft-tissue thickness in elective total hip replacement surgery. A case-control study was conducted to compare the soft-tissue thickness radiographic measurement (SRM) at the hip in patients diagnosed with APJI based on Tsukayama et al. (2003) criteria after total hip replacement with patients that were not infected, at a single institution from 2013 to 2019. To minimize selection bias, each case was matched with two controls using the following methodology: patients of the same sex, with an age variation of ± 5 years, and nearest in surgery date to the cases were selected. All postoperative radiographs were performed in the first 24 h after total hip arthroplasty (THA) surgery as it is protocolized in our institution. Soft-tissue thickness radiographic measurement was defined as the distance from the tip of the greater trochanter to the skin following a perpendicular line to the femoral diaphysis in postoperative anteroposterior hip radiographs. In total, 78 patients were included (26 cases and 52 controls). The SRM median of the cases was 76.19 mm (SD: 26.518) and 53.5 mm (SD: 20.47) in controls. A multivariate logistic regression model showed an independent association between APJI and SRM (odds ratio (OR) = 1.033, 95 % confidence interval (CI) 1.007–1.059, p=0.012). Patients with an SRM greater than 60 mm had a 7-fold increase in the odds of APJI (OR = 7.295, 95 % CI = 2.364–22.511, p<0.001). The results of our study suggest an association between large SRM at the hip and the risk of APJI in patients with primary total hip arthroplasty. SRM may be a helpful and easy tool for evaluating the risk of APJI before elective primary total hip replacement surgery.

## 1 Introduction

Acute periprosthetic joint infection following total hip arthroplasty (THA) can dramatically modify a patient's postoperative expectations, increasing the challenge of postoperative management, sometimes even requiring revision surgery to eradicate infection and reconstruct a functional hip (Triantafyllopoulos et al., 2015).

APJIs not only cause an important adverse impact on patient outcomes but also in healthcare economic systems. Prevalence estimations have predicted an increase in total hip replacement procedures in future years, and it is fitting to assume an increase in APJI.

Host susceptibility to infection has emerged as an important predictor of APJI. Several risk factors for acute postoperative wound complications and infection following THA have previously been identified. These include diabetes, immunosuppression, inflammatory arthropathy, primary bone malignancy, prior lower-extremity fracture, renal or hepatic diseases, age, alcohol, parenteral drugs or tobacco abuse, vascular insufficiency, and obesity (Wright et al., 2012).

Although preoperative risk assessment is multifactorial, radiographically measured soft-tissue thickness at the incision site has been associated with postoperative surgical site infection after cardiac, cervical spine, and lumbar spine surgery (Kozlow et al., 2014; Lee et al., 2016; Mehta et al., 2013). Also, computerized-tomography-measured abdominal soft-tissue depth has been correlated in abdominal and spine surgery (Fuji et al., 2010; Lee et al., 2011).

Increased anterior knee subcutaneous fat thickness measured in radiographs has been associated with a significantly increased risk of early reoperation for wound complications following primary total knee arthroplasty (TKA) and had a greater predictive value than body mass index (BMI) (Watts et al., 2016; Wagner et al., 2018). However, few studies have investigated the potential association between increased peritrochanteric soft-tissue thickness and APJI following primary THA.

The exact measurement of soft-tissue thickness can only be assessed intraoperatively. For this reason, preoperative detection of patients at a higher risk of acute periprosthetic joint infection is challenging, and preventive strategies are limited.

The objective of our study was to evaluate the association between acute periprosthetic joint infection (APJI) and soft-tissue thickness measured with an X-ray after elective total hip replacement surgery following the methodology previously described by Bernaus et al. (2019).

This study hypothesizes that large measurements of local soft tissue around the hip, measured with radiographs, are associated with an increased risk of APJI after total hip replacement.

## 2 Materials and methods

A case-control study was conducted to compare soft-tissue thickness radiographic measurement (SRM) at the hip in patients diagnosed with APJI after total hip replacement with patients that were not diagnosed with APJI. For this study, we included all patients diagnosed with APJI admitted to our hospital for total hip replacement from 2013 to 2019. All the patients were diagnosed with hip arthritis.

All patients were operated on by the same surgical team (Francesc Angles Crespo, Agustí Bartra Ylla, and Silvia Maria Miguela Álvarez) from the Hip Unit at our institution. Antibiotic prophylaxis dosing adjusted by weight was performed with a second-generation cephalosporin for non-allergic patients, clindamycin for allergic patients, and teicoplanin for patients with colonization with methicillin-resistant *Staphylococcus aureus*. A posterolateral approach was performed in all cases. No drains were used.

All patients were included in a fast-track program with mobilization on the same day of the surgery.

### 2.1 Patients and variables

Cases were defined as patients diagnosed with APJI (those diagnosed less than 4 weeks after surgery) after total hip replacement. To minimize selection bias, each case was matched with two controls using the following methodology: patients of the same sex, with an age variation of ±5 years, and nearest in surgery date to the cases were selected. All participants had high-quality anteroposterior pelvis X-rays available. The principal variables collected were soft-tissue thickness radiographic measurement and acute periprosthetic joint infection. Secondary variables collected included age; gender; BMI; operating time; American Society of Anesthesiologists (ASA) physical status classification; and comorbidities such as diabetes mellitus, rheumatoid arthritis, transfusion, liver cirrhosis, current use of corticosteroids, smoking, and parental drug use.

### 2.2 Criteria for APJI diagnosis

The clinical diagnosis of acute periprosthetic joint infection (APJI) was based on Tsukayama et al. (2003) criteria: local inflammatory signs, purulent drainage through the wound, and elevated C-reactive protein during the first 4 weeks after the index surgery.

### 2.3 Surgical technique and antibiotic therapy

Surgical debridement was performed in all patients with a clinical diagnostic suspicion of APJI. During the surgery, at least five to seven different samples were taken to obtain a microbiological diagnosis, and modular components were changed. Synovial fluid samples were inoculated in blood culture flasks (Font-Vizcarra and Soriano, 2010). After surgery, broad-spectrum intravenous antibiotic therapy with antibiofilm activity was started following the protocol at our institution: teicoplanin, amikacin, and rifampicin until results from intraoperative cultures were available. Then, targeted antibiotic therapy was started according to the antibiotic sensibility of the specific microorganism for 8 weeks. Oral medication was preferred when possible.

### 2.4 Methodology for assessment of SRM

**Figure 1:**
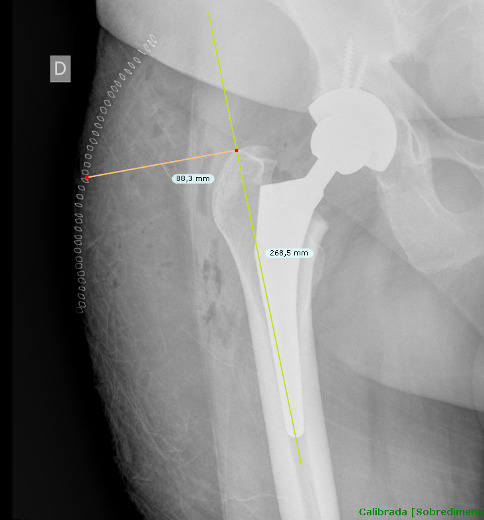
The distance from the tip of the greater trochanter to the skin was measured following a perpendicular line to the femoral diaphysis in anteroposterior hip radiographs.

**Figure 2:**
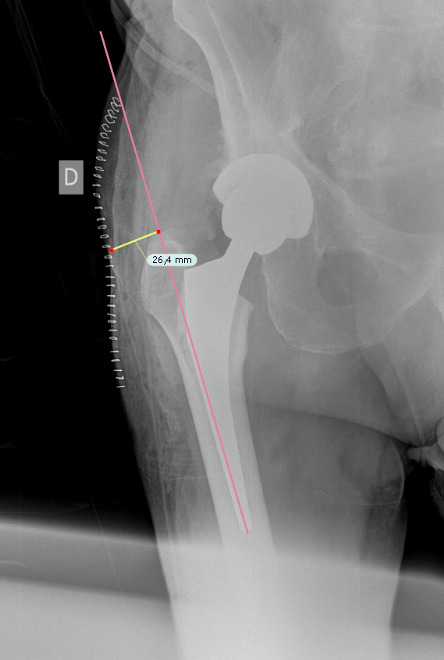
SRM, in this example, is 26.4 mm.

Once cases and controls were identified and included, the distance from the tip of the greater trochanter to the skin was measured by two different blinded orthopedic surgeons following a perpendicular line to the femoral diaphysis in anteroposterior hip radiographs. Standardized anteroposterior pelvis X-rays (Figs. 1 and 2) in the supine position and 15${}^{\circ }$ of internal rotation of the hips were obtained for all patients.

To obtain precise measurements, the first step required calibrating X-rays. In patients with THA, the known diameter of the dome/cup component was introduced into the TraumaCad X-ray viewer program (Brainlab Ltd., Petach Tikva, Israel) and set as a reference. Once this calibration process was performed, we proceeded to obtain the measurement from the tip of the greater trochanter to the skin as previously described. These measurements were taken in the postoperative X-ray performed in the first 24 h after THA.

### 2.5 Statistical analysis

Qualitative or ordinal variables were described using absolute frequencies or percentages while quantitative variables were described as mean or median values when necessary. Univariate analyses were performed using the Student's T test, Fisher test, or chi-squared test for quantitative and dichotomous variables. SRM was analyzed as a continuous variable and as a dichotomous variable using the median of our measurements as a threshold. We performed a multivariate logistic regression model with those significant variables in univariate analysis. A multivariate logistic regression model was used to estimate the risk of presenting infection as an odds ratio (OR) and with a 95 % confidence interval (CI). All analyses were performed using SPSS for MAC v22 (SPSS Inc., Chicago, IL, USA). The level of statistical significance was established at p≤0.05 (two-tailed).

## 3 Results

A total of 81 patients (27 cases and 54 controls) were included in this analysis. Three patients had to be excluded, two controls (women) and one case (man), as the postoperative X-ray did not include the skin. From those 78 patients (26 cases and 52 controls), there were 31 (39.7 %) female patients and 47 (60.3 %) male patients. The mean age of APJI patients was 67 years (SD = 12.7) and 68.54 years (SD = 12.4) for non-APJI patients.

The microorganisms isolated were coagulase-negative *Staphylococci* (8 cases, 29.63 %), methicillin-sensitive *S. Aureus* (MSSA) (7 cases, 25.97 %), Gram-negative rods (5 cases, 18.5 %), mixed flora (4 cases, 14.8 %), ciprofloxacin-sensitive *Pseudomonas aeruginosa* (1 case, 3.7 %), *Enterococcus faecalis* (1 case, 3.7 %), and *Enterobacter cloacae* (1 case, 3.7 %).

In the univariate analysis, the distribution of previously studied risk factors between cases and controls did not show significant differences except for soft-tissue thickness radiographic measurement, patients with an ASA score of 3, operating time, and BMI (Table 1).

**Table 1:** Univariate analysis results.

		Infection		P value
	No (n=52)	Yes (n=26)	Total	
Mean age in years (SD)	68.54 (12.388)	67.23 (12.697)	68.1 (12.4)	0.664
Female gender	21 (40.4 %)	10 (38.5 %)	31 (39.7 %)	1
Diabetes mellitus	8 (15.4 %)	6 (23.1 %)	14 (17.9 %)	0.533
Smokers	8 (15.4 %)	4 (15.4 %)	12 (15.3 %)	1
ASA score 1	4 (7.7 %)	1 (4 %)	5 (6.5 %)	0.138
ASA score 2	33 (63.5 %)	11 (44 %)	44 (57.1 %)	0.138
ASA score 3	15 (28.8 %)	13 (52 %)	28 (36.4 %)	0.076
Rheumatoid arthritis	2 (3.8 %)	2 (7.7 %)	4 (5.1 %)	0.597
Liver cirrhosis	1 (2 %)	1 (3.8 %)	2 (2.6 %)	1
Use of corticoids	3 (5.8 %)	1 (3.8 %)	4 (5.1 %)	1
Mean soft-tissue thickness radiographic measurement in millimeters (SD)	53.5 (20.47)	76.19 (26.518)	61.06 (24.93)	< 0.001
Operating time in minutes (SD)	68.05 (68.05)	83.2(83.20)	73.7 (20.08)	0.01
Mean BMI (SD)	28.09 (4.76)	32.20 (6.07)	29.46 (5.55)	0.002

SD: standard deviation.

A correlation study resulted in a positive correlation between the variables that showed an association in the univariate model (operating time, BMI, and SRM). Operating time and SRM showed a weak uphill (positive) linear relationship (Pearson correlation coefficient = 0.4). BMI and operating time showed a weak uphill (positive) linear relationship (Pearson correlation coefficient = 0.3). BMI and SRM showed a moderate uphill (positive) relationship (Pearson correlation coefficient = 0.561). All correlations showed statistical significance.

Finally, we performed a multivariate logistic regression model to investigate the association between APJI and variables that showed association in the univariate model (operating time, BMI, and SRM). This analysis showed an independent association between APJI and SRM (OR = 1.033, 95 % CI 1.007–1.059, p=0.012). The other variables analyzed (operating time and BMI) were progressively excluded from the equation when the Wald method was used.

The median for the SRM at the hip for all patients was 60 mm (range 15–141). The mean SRM for cases was 76.19 mm (SD = 26.52) and 53.5 mm (SD = 20.47) for controls. The median for the case group was 79 mm (15–141) and 48.5 mm (25–128) for the control group. When participants were separated into two groups based on the median as a threshold, a significant association between APJI and SRM was found for patients with greater measurements. Patients with an SRM greater than 60 mm had a 7-fold increase in the odds of APJI (OR = 7.295, 95 % CI = 2.364–22.511, p<0.001) compared to those with smaller measurements.

A subanalysis based on different sex was performed to test the correlation between BMI and SRM. We expected a different fat distribution between sexes but the correlation between BMI and SRM was still present. This may be explained by the inclusion of muscle and not only subcutaneous fat in the measurement.

## 4 Discussion

Since the appearance of total joint arthroplasty, antibiotic prophylaxis and improvements in surgical technique have reduced APJI rates. However, with an increasing number of yearly total hip replacements, a substantial population remains at risk of infection-related complications following this procedure.

Accurate preoperative infection-risk stratification is an important step toward further reducing APJI rates. In this way, surgeons could thoroughly discuss with their patients the infection risk of surgery, possible non-surgical alternatives, and especially possible means of risk reduction.

A non-invasive tool for detecting those patients at increased risk of APJI could allow additional prevention strategies. Some studies have determined that infection risk can be objectively determined in a preoperative setting with an APJI risk-assessment score attending to patient comorbidities (Everhart et al., 2016; Poultsides et al., 2018), but these do not take into account local conditions around the joint.

Multiple explanations can be considered as to why an increased local fat distribution at the hips is associated with an increased risk of APJI. Having an increased physical space implies technical difficulties for surgeons leading to larger dissections, soft-tissue damage, aggressive retraction, an increased surgical time, and subsequently a higher risk of infection.

The prevalence of obesity continues to rise in parallel with the demand for total hip arthroplasty (Pirruccio et al., 2019). A relationship between surgical site infection (SSI) and morbid obesity or diabetes has consistently been reported; although both conditions are often concurrent, both diabetes and morbid obesity independently contribute to SSI risk. Moreover, obesity has previously been demonstrated to be an independent risk factor for increased complications after total hip and knee arthroplasties (Dowsey and Choong, 2008; Ma et al., 2016; Chee et al., 2010; Zingg et al., 2016).

BMI is also known to be a predictive factor for wound complications following both THA and TKA (Wallace and Judge, 2014; Shearer et al., 2020). Adhikary et al. (2016) suggested that there was a positive correlation between BMI and incidences of 30 d postoperative complications in both TKA and THA.

Radiographic measurement of soft-tissue thickness has been correlated with postoperative surgical site infection in other surgical fields such as general and cardiac surgery (Kozlow et al., 2014; Lee et al., 2011).

In general surgery, Lee et al. (2011) found that abdominal subcutaneous fat measured in CT is an independent predictor of superficial incisional SSI after midline laparotomy, and Fuji et al. (2010) also reported an increased risk of SSI in patients with greater radiographically determined subcutaneous fat thickness after undergoing elective colorectal resection.

A similar study on elective lumbar spine surgery shows the utility of local soft-tissue thickness radiographic measures as a simple independent risk assessment tool for surgical site infection (Lee et al., 2016). In cervical spine fusion procedures, Mehta et al. (2013) demonstrated that the thickness of subcutaneous fat measured by CT scan is a factor in the development of surgical site infection.

DeMik et al. (2018) established that the impact of obesity on postoperative complications is more profound for THA than TKA. Morbidly obese patients undergoing THA were also found to have significantly higher rates of wound complications, deep infection, and reoperation rate. Moreover, DeMik took into account the fat distribution differences between the knee and hip soft tissues. The tendency of adipose tissue to deposit in the gluteal region and the resulting soft-tissue envelope may contribute to the differences between THA and TKA postoperative complications.

All hip arthroplasties in our study were implanted using a posterolateral approach. A recent study by Purcell et al. (2018) found there was no difference in deep infection rates between a direct anterior and posterior approach.

In a series of morbidly obese patients undergoing primary TKA, Watts et al. (2016) found increased anterior knee subcutaneous fat thickness using plain radiographs to be a more predictive measurement of wound complication than body mass index. In a recent study, Shearer et al. (2020) found that BMI is a better predictor of periprosthetic joint infection risk than radiographic measures of adipose tissue after TKA, as opposed to Watts. In our study, 4 patients had a normal BMI with > 60 mm SRM. It is important to take into consideration that fat distribution is different between the knee and hip when comparing these results.

The impact of SRM in elderly patients with surgically treated hip fractures was previously studied (Bernaus et al., 2019). The analysis demonstrated an association between the SRM at the hip and the risk of SSI. They found that patients with an SRM greater than 6.27 cm had a 7-fold increase in the odds of surgical site infection compared to those with smaller measurements. In the same way, patients with infection had a 2.24 cm greater mean SRM.

Increased peritrochanteric soft-tissue thickness following primary total hip arthroplasty and its association with APJI has been barely studied. Bell et al. (2019) performed a case-control study matched on age, sex, and BMI which did not demonstrate an association between radiographically measured peritrochanteric fat and infectious or wound complications following THA. They also performed the surgery using a posterior surgical approach. Peritrochanteric fat thickness was reliably measured, with Pearson's correlation coefficients > 0.90 in all cases. Also, Mayne et al. (2020) analyzed 1220 primary THAs and measured, intraoperatively, the vertical soft-tissue depth from the most prominent part of the greater trochanter to the skin. Surgery was performed through a posterior approach as we did in our study. They did not find a relationship between peritrochanteric fat depth and the risk of surgical complications for the initial 12-month follow-up period. Patients with BMI > 40 kg/m${}^{2}$ or more had a significantly increased risk of infection compared to those with BMI < 40 kg/m${}^{2}$. They also found females had a significantly greater fat depth at the greater trochanter in comparison to males, although no significant differences in BMI were observed between sexes. Fat depth showed a weak correlation with BMI.

The results of our study suggest an association between the SRM at the hip and the risk of APJI in patients after total hip replacement surgery. Sprowls et al. (2020a) retrospectively reviewed 1110 patients, identifying increased lateral soft-tissue thickness and body mass index as factors associated with surgical site infection and deep infection and lateral soft-tissue thickness with revision surgery as well. A value > 5 cm was predictive of surgical site infection, deep infection, and revision surgery. All surgeries were performed through a lateral incision (posterior, lateral, or anterolateral approach). The measurement of lateral soft-tissue thickness was measured as the horizontal distance from the most lateral point of the greater trochanter to the skin edge, using the standing hip radiographs obtained within 1 year of the surgery date. Increased lateral soft-tissue thickness was associated with risk for revision surgery (OR 1.02 per increasing millimeter).

The same group (Sprowls et al., 2020b) examined the relationship between fat thickness and 90 d postoperative complications and assessed the intraoperative thickness of subcutaneous fat at the incision site for direct anterior and posterior approaches for THA. In total, 124 patients were included and reviewed retrospectively. Also, the lateral hip fat thickness was measured from preoperative anteroposterior pelvis radiographs (taken within 6 months of the THA procedure). Return to the operation room was significantly associated with BMI, anterior soft-tissue incision site, and lateral soft-tissue incision site. Periprosthetic joint infection (American Academy of Orthopaedic Surgeons (AAOS) diagnostic criteria) was significantly associated with BMI and lateral incision site fat thickness. Lateral hip fat thickness measured in radiographs strongly correlated with measurements at the incision site. Regardless of BMI, sex, or age, more soft tissue was encountered with posterior approaches compared to direct anterior approaches. Excess incisional fat was associated with periprosthetic joint infection after a posterior approach.

Likewise, our multivariate logistic regression model showed an independent association between APJI and SRM (OR = 1.033, 95 % CI 1.007–1.059). Patients with an SRM greater than 60 mm had a 7-fold increase in the odds of APJI (OR = 7.42, 95 % CI = 3.01–18.28, p<0.001) compared to those with smaller measurements. The reliability of measurements using this method between observers was previously corroborated by Bernaus et al. (2019). The overall agreement between observers was good (intraclass correlation coefficient score = 0.822, 95 % CI: 0.754–0.873, p<0.001).

There are some limitations in this study: it includes a small sample size from a single institution that may compromise external validity, and for this reason, we believe there is a need for further study in this area of interest.

Postoperative radiographs were used for the measurement of distances as 25 % of calibrated preoperative radiographs did not include the skin. Three patients had to be excluded as the postoperative X-ray did not include the skin. Postoperative inflammation and hematoma formation can impact the soft-tissue thickness. However, hematoma formation is not an important issue with the use of tranexamic acid.

All radiographs were taken in the first 24 h after THA surgery, as it is protocolized in our institution. These radiographs were taken in a supine position which could influence exact measurements. Some potential drawbacks to the SRM could include different patient positioning, rotation of the pelvis, and the orientation of the beam.

Contrary to other previously reported studies (Bell et al., 2019; Mayne et al., 2020), radiographic measurement of soft-tissue thickness was a valid tool for predicting APJI after primary total hip arthroplasty in this study. This measure allows for long-term individualized prevention strategies to decrease the soft tissue around the hip, especially in elective patients in which time to surgery is not critical. This measurement could alert about higher infection risk in patients with normal BMI values or without systemic risk factors.

Prevention strategies, including preoperative weight control (which could also decrease osteoarthritis symptoms), optimization of antibiotic prophylaxis doses, the use of local antibiotics, or povidone iodine in cases that preoperatively prove to be at higher risk, could be established as a clinical routine.

## 5 Conclusions

The results of our study suggest an association between large SRM at the hip and the risk of APJI in patients with primary total hip arthroplasty. SRM may be a helpful and easy tool for evaluating the risk of APJI before elective primary total hip replacement surgery. However, further studies are necessary to confirm our results and to further evaluate its usefulness in clinical practice.

## Data Availability

Research data are not published but can be requested by email to the corresponding author.
